# A Combined Independent Source Separation and Quality Index Optimization Method for Fetal ECG Extraction from Abdominal Maternal Leads

**DOI:** 10.3390/s17051135

**Published:** 2017-05-16

**Authors:** Lucia Billeci, Maurizio Varanini

**Affiliations:** Institute of Clinical Physiology, National Research Council of Italy, via Moruzzi 1, 56124 Pisa, Italy; maurizio.varanini@ifc.cnr.it

**Keywords:** fetal ECG extraction, abdominal ECG, independent component analysis (ICA), quality index, optimization

## Abstract

The non-invasive fetal electrocardiogram (fECG) technique has recently received considerable interest in monitoring fetal health. The aim of our paper is to propose a novel fECG algorithm based on the combination of the criteria of independent source separation and of a quality index optimization (ICAQIO-based). The algorithm was compared with two methods applying the two different criteria independently—the ICA-based and the QIO-based methods—which were previously developed by our group. All three methods were tested on the recently implemented Fetal ECG Synthetic Database (FECGSYNDB). Moreover, the performance of the algorithm was tested on real data from the PhysioNet fetal ECG Challenge 2013 Database. The proposed combined method outperformed the other two algorithms on the FECGSYNDB (ICAQIO-based: 98.78%, QIO-based: 97.77%, ICA-based: 97.61%). Significant differences were obtained in particular in the conditions when uterine contractions and maternal and fetal ectopic beats occurred. On the real data, all three methods obtained very high performances, with the QIO-based method proving slightly better than the other two (ICAQIO-based: 99.38%, QIO-based: 99.76%, ICA-based: 99.37%). The findings from this study suggest that the proposed method could potentially be applied as a novel algorithm for accurate extraction of fECG, especially in critical recording conditions.

## 1. Introduction

Fetal heart rate (FHR) monitoring during pregnancy is clinically relevant and can be obtained with several invasive or non-invasive techniques, including Doppler ultrasound fetal magneto- cardiography (FMCG) and fetal electrocardiography (FECG). Doppler ultrasound is the traditionally applied technique for monitoring the fetus during pregnancy. It can usually identify and measure embryonic heartbeat by six weeks [1], while exams performed between 18 and 22 week of gestation allow screening for most fetal cardiac anomalies [2]. In particular, congenital heart disease (CHD) is the most common congenital anomaly worldwide [3], with an incidence estimated at 6–12 cases per 1000 live births [4]. Early diagnosis of CHD during pregnancy can increase survival rates of the fetus and decrease long-term morbidity in both ductus-dependent and foramen ovale-dependent CHD [5]. However, the probability of ultrasound to accurately detect CHD ranges from 65% to 81%, with a significant part of events missed. Inaccuracies in ultrasound detection can be due to the complex anatomy of the fetal heart, its movement, small size and mixing maternal and fetal heart rate. Higher detection rates can be achieved using three- and four-dimensional ultrasonography [6]. The disadvantages of these techniques are that they require expert personal and that they are extremely expensive. FMCG can be recorded reliably from the 20th week onward. Compared to ultrasound it has a higher resolution and higher signal quality, allowing an assessment of PQRST complex alterations, and detecting fetal arrhythmia. Early diagnosis of fetal arrhythmia permits an appropriate therapeutic intervention and the reduction of unexplained fetal death in late gestation [7]. 

Compared to ultrasound and FMCG, fetal electrocardiography (fECG) is more cost-effective and provides additional useful information for an accurate evaluation of the fetal status, having the potential to provide FHR data with beat-to-beat accuracy. fECG can be performed invasively using *intra-uterine* electrodes, which have a direct contact with the scalp of the fetus. Despite providing a high quality fECG, this technique has consistent drawbacks due to its invasive nature and its limited applicability during labor. Recently, non-invasive fECG recording has received considerable interest in monitoring fetal health. With this modality, signals are recorded by multiple electrodes placed on the abdomen of the mother. Non-invasive fECG has numerous advantages including the safeness, the possibility of long-term continuous monitoring, the wide time-range of applicability (from 18 weeks of gestation) and the relative low cost. On the other hand, several technical challenges prevent a direct usability of the acquired signals. In particular, the fetal signal has low amplitude and is mixed with several sources of noise and interference [8] such as maternal ECG (mECG), baseline drifts, power line, muscle electrical activity (EMG), maternal respiration, motion artefacts and electrode contact noise. These sources of noise often have higher amplitude than the fECG so the signal to noise ratio (SNR) is low. Fetal ECG amplitude can vary depending on several factors of the recording setup. For example, skeletal muscle artifacts introduce high frequency components between 10 Hz and 500 Hz during skeletal muscle activity, in particular during a contraction, masking fECG. In addition, fetal movements can result in a different orientation of the fetal heart vector with respect to the electrode grid, changing the amplitude and morphology of the measured signals. In addition, fECG often overlaps in time and in frequency with mECG and the other noise components so the extraction of fECG from abdominal leads results in a very challenging task. Importantly, it should be noted that between the 28th and 32th weeks of gestation the recording of surface fECG is unfeasible due to the formation of the *vernix caseosa*, a thin fatty layer which almost electrically shields the fetus [9]. However, for normal pregnancies (non-premature deliveries), the layer slowly dissolves in the 37th to 38th weeks of pregnancy [10].

For these reasons, different signal-processing methods have been implemented to extract fECG from abdominal mixtures (for a review see [11]). Methods for fECG extraction can be broadly divided into two groups: mECG canceling and blind source separation (BSS). The first group includes the regression-based methods which use the mECG as reference input to estimate and cancel its contribution on abdominal signals. This task can be performed in a continuous way by adaptive filtering (AF) or in a beat-by-beat mode by template subtraction (TS). AF [12,13] uses maternal reference channels to estimate their projection onto each abdominal signal [13,14,15,16]. TS can be considered as a special case of AF that uses as reference input signal an impulse sequence synchronized with maternal beats [12,17]. However, TS is usually implemented by first estimating the mean contribution of the maternal cycle (template) and then subtracting it from the mixture of abdominal signals [18,19]. A powerful method to estimate the contribution of each single maternal heart beat to the abdominal signal is to use a reduced space approximation by Principal Component Analysis (PCA) which can be implemented by Singular Value Decomposition (SVD) [20,21,22,23].

BSS aims at separating the different components of the abdominal mixture without a priori knowledge of the signal, but according to the statistical properties of the data. Commonly used approaches are PCA or SVD [24,25] and Independent Component Analysis (ICA) [26,27,28].

Hybrid methods consisting in a combination of the previously described methods have also been developed. Some approaches have been implemented, for example combining TS and BSS [29,30].

Recently our research group has contributed to the challenging task of extracting fECG from abdominal maternal signals by developing two different signal-processing methods. Our first algorithm was developed for the PhysioNet/Computing in Cardiology Challenge 2013 (CinC 2013), which promoted the development of accurate and robust algorithms for estimating fHR, fetal interbeat intervals, and fetal QT intervals from multichannel maternal ECG recordings. Further details about the CinC 2013 can be found in [31], which discussed the background issues, the design of the Challenge, the key achievements, and the follow-up research. This first algorithm that we developed, called the ICA-based method, belongs to the hybrid group of methods, being based on the sequence of ICA, mECG canceling (in particular TS) and a second ICA [23] and obtained the top official scores during event 1 and event 2 concerning fetal heart rate and fetal interbeat intervals estimation section [32]. However, there was one non-official higher score, as reported in [31], obtained by Behar and colleagues [33], which also implemented a hybrid method based on the fusion of several different techniques of source separation (including PCA, template subtraction, and ICA).

Despite the high performance of our first algorithm, for a few records it failed in extracting a sufficiently clean fECG. This drawback could be ascribed to the model order selection problem. In theory, the number of independent sources is undetermined and always higher than the number of acquired signals, thus ICA method works in a sub-optimal contest and it will be able to separate the independent sources of interest only if their components in the acquired mixed signals have higher power greater than the others. To face this problem, we implemented a second algorithm, the quality index optimization (QIO)-based method, which a priori takes into account specific characteristics of the fECG rather than only the unspecific independence of sources [34]. In this second method, two quality indexes (fQI and mQI) were devised, which discriminate the two components of interest (fECG and mECG, respectively) from noise sources. These indexes were built exploiting the morphological and temporal characteristics of the fECG/mECG signals. An optimization procedure based on the Nelder-Mead algorithm was then applied in order to find a linear combination of abdominal signals, which maximizes these indexes. This method can be considered a novel approach for fECG extraction, which attempt to find the fetal QRS (fQRS) in the abdominal mixture by exploiting its characteristics. 

In our recent paper [34] this QIO-based method was compared with the ICA-based one on the same dataset extracted from the PhysioNet Challenge 2013 Database and it outperformed the ICA-based approach for most of the records. 

However, when comparing the record-by-record performance of the ICA-based and QIO-based methods we observed that, although generally the QIO-based method outperformed the ICA-based one, for some records, the opposite was true [34]. Thus, we hypothesized that a combination of the two approaches could improve the performance in fQRS detection and reduce the number of records with low performance. Indeed, as the QIO-based method and the ICA-based approach use different information in separating fECG, an integration of the two criteria should lead to an improvement in the performance in fECG extraction. In this paper, we propose a novel method, which combines both the criteria: that based on the independence of sources and that based on the a-priori specific characteristics of sources. In the following section the algorithm proposed in this paper will be referred as “ICAQIO-based” method.

Recently a Fetal ECG Synthetic Database (FECGSYNDB) for benchmarking of fetal ECG extraction and detection algorithms has been developed [35]. Data were generated using the fetal ECG synthetic simulator (*fecgsyn*, [36]), which can generate maternal-fetal ECG mixtures with realistic amplitudes, morphology, beat-to-beat variability, heart rate changes and noise. In [35] the authors also evaluated their methodology by testing some common fECG extraction methods on the developed database. Successively another research group has used the FECGSYNDB to evaluate the performance of its fECG extraction algorithm based on sequential total variation denoising [37].

The aim of our paper is to propose the novel “ICAQIO-based” method and evaluate its performance on the recently developed FECGSYNDB as well as on the PhysioNet CinC 2013 Database, on which we already tested our previously developed algorithms. In order to test the improvement achieved by the combination of the independence of sources and the quality index optimization with the performances obtained applying the two criteria separately, we compare the performances of the “ICAQIO-based” method with that of the “ICA-based” and “QIO-based” approaches.

## 2. Materials and Methods

### 2.1. Data

The FECGSYNDB on which algorithms were tested consists of 1750 synthetic signals. The database is structured in seven cases resembling different physiological events (see Table 1) and for each case five different levels of additive noise are included (0, 3, 6, 9, and 12 dB). In addition, for each case ten different heart dipole models were generated by randomly selecting one of the nine vectorcardiograms available in the fecgsyn toolbox. Finally, simulations were repeated five times to obtain a more representative database. Each simulation is 5 min long for a total of 145.8 h, it is sampled at 250 Hz with a 16-bit resolution and it is projected onto 34 channels (32 abdominal and two mECG reference channels). For details about the parameters used for the simulation see [35,36].

In order to compare the performance of our algorithms with that of the ones already tested on the same database, we selected the same combination of channels of the previous study [35]. In particular, among the different combinations used, we selected the one with 4 channels (1, 11, 22 and 32). This choice was motivated by the fact that it allows ICA to achieve the separation of sources without loading excessively the mother with abdominal channels. In addition, the performance of the Nelder-Mead algorithm improves in low dimension [38]. We also selected channels 33 and 34, which are the mECG reference channels. It should be noticed that, compared with the PhysioNet CinC 2013 Database, in the FECGSYNDB there are reference mECG channels, so the algorithms do not include the mECG extraction step as our previous implementations [23,34]. 

Among the seven cases of the database we discard case 5 as the QIO-based approach is not suitable to detect more than one fECG as it will be better explained in the following paragraphs. Moreover, we selected only the two highest levels of noise (i.e., SNR = 0 and SNR = 3 dB) as, according to previous results [35,37], they bring out the most significant differences among the different algorithms.

To test the performance of the proposed method on real data, we also used the annotated open set of recordings “set-a” of the PhysioNet CinC 2013 Database. The dataset consists of 75 records (length: 60 s) from five abdominal signal collections. Each record includes four channels of maternal abdominal ECG sampled at 1 KHz. Further details about the PhysioNet CinC 2013 Database can be found in [31,39]. The records a33, a38, a52, a54, a71, a74 were excluded because they had partial or inaccurate reference annotations. Moreover, the first and the last annotated beat of each record were ruled out from the evaluation because their reference annotations were often inaccurate. 

### 2.2. Tested Methods

**Figure 1:**
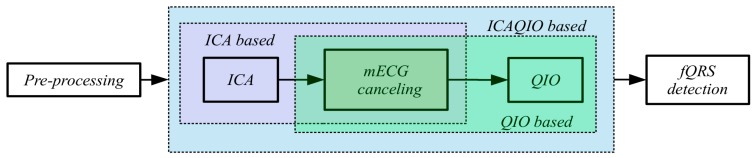
Block-diagram of the three methods compared in this study: ICAQIO-based, QIO-based and ICA-based. The pre-processing and the fQRS detection steps are common to the three algorithms.

In this paper, we tested the novel proposed “ICAQIO-based” method and we compared it with our previously developed “ICA-based” and “QIO-based” approaches. The three different methods are schematized in Figure 1.

The three extraction algorithms that are differently aggregated in these methods are: ICA, mECG canceling and a QIO algorithm. ICA, which is a form of BSS technique assuming the independence of sources, is becoming a very widely applied method in fECG. Maternal ECG canceling is one of the most commonly applied technique for fECG extraction and in this study, it is implemented as TS. Conversely, the third algorithm, was first introduced by our group in [34].

In Section 2.3 we will fully describe the “ICAQIO-based” method, which we propose in this paper (Figure 1). In Section 2.4, we will briefly describe the “ICA-based” and “QIO-based” approaches (Figure 1), for a full description see [23,34], respectively.

### 2.3. Proposed Combined Method: ICAQIO-Based

The proposed method attempts to extract the fECG from the abdominal mixture on the basis of the combination of our previous approaches, respectively based on ICA [23] and on the optimization of a quality index built on the morphological and temporal characteristics of the signal [34]. As the two methods are based on two different criteria, we figured out that their combination could outperform each single method. The proposed ICAQIO-based method includes five steps: pre-processing, separation of sources based on ICA, maternal ECG canceling, enhancement of fQRS based on fQI optimization and fQRS detection (Figure 1). The main steps of the algorithm are summarized hereafter, for a detailed description see [23,34].

#### 2.3.1. Pre-Processing

**Figure 2:**
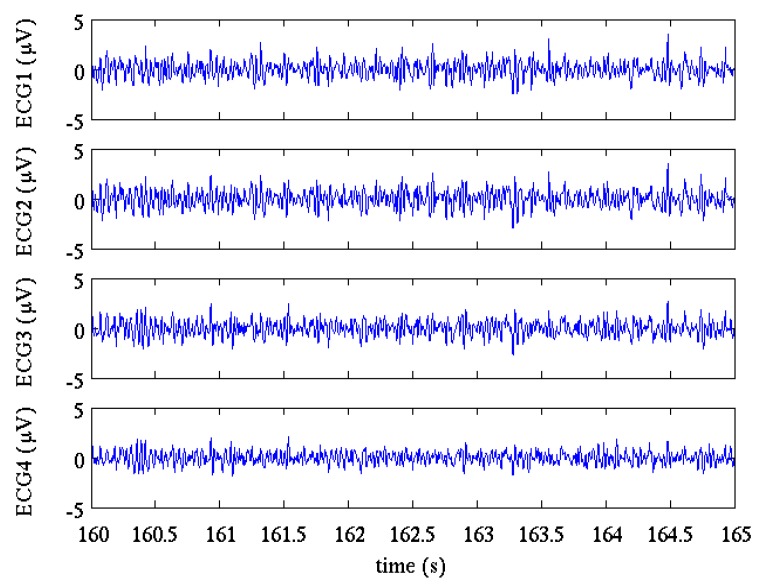
5 s interval of the four selected channels of the FECGSYNDB after pre-processing. Selected record: dipole model = 2, simulation = 1, case = 3, SNR = 0 dB.

Pre-processing aimed at removing the most undesired noisy components before separating mECG and fECG. The same pre-processing steps were applied for all the three methods tested in these studies and include: impulsive artefacts canceling, baseline wandering removal and power-line interference canceling. A detailed description of the procedures used in this study can be found in our previous papers [23,34]. Figure 2 shows a 5 s interval of the four selected channels of the FECGSYNDB after pre-processing.

#### 2.3.2. Independent Component Analysis 

The application of ICA after pre-processing aims at separating the fECG from the mECG and the other components. These components include the electromyographic signal, residual noise and artefacts. Among the different approaches proposed for fECG extraction, BSS is one of the most commonly applied [24,25,26,27,28,29,40]. It attempts to decompose the multichannel abdominal mixture into the different components i.e., mECG, fECG and noise. BSS can be performed using PCA, which assumes that the signals are a linear combination of the sources, that large variance represents interesting structures and that the principal components are orthogonal. However, the second assumption could not be satisfied, which means the maximization of variance criterion does not comply with fECG, mECG and noise source separation. Conversely ICA, beyond the linear mixing, assumes that the sources are statistically independent, non-Gaussian and/or autocorrelated; assumptions that are generally satisfied for fECG, mECG and noise sources. 

**Figure 3:**
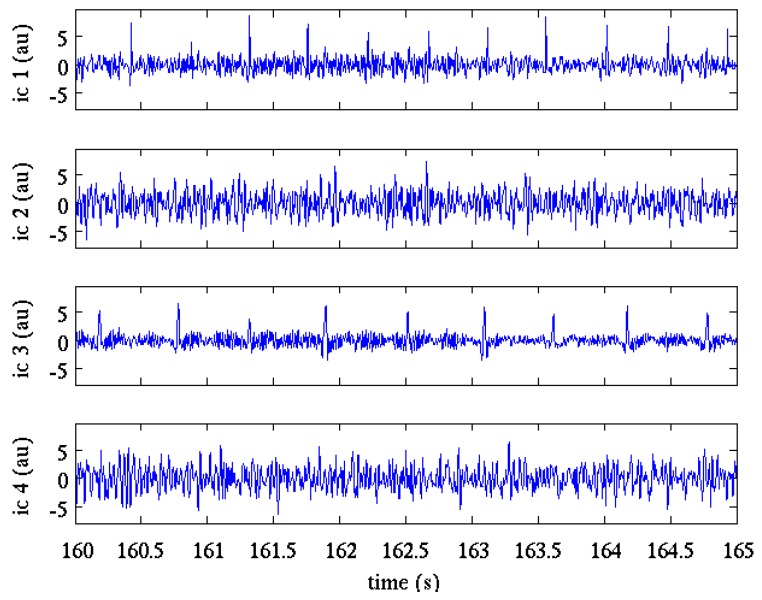
Components resulting from ICA application to the signals in Figure 2. Intensity values are expressed in arbitrary units (au).

Several algorithms have been implemented, which realize ICA, including second order blind identification (SOBI) [41], joint approximate diagonalization (JADE) [42] and FastICA [43]. In our approach, the FastICA [43] with deflationary orthogonalization was selected as ICA algorithm as it gave the most reliable results respect to the other tested algorithms (SOBI and JADE) [23]. The ICA algorithm was applied using the all registration length as block size. The hyperbolic cosine was the preferred contrast function; in the few cases when convergence failed, kurtosis was automatically selected. Figure 3 shows the results of ICA applied to the pre-processed signals shown in Figure 2.

#### 2.3.3. Maternal ECG Cancelling

After the separation of the components constituting the abdominal mixture, mECG canceling was applied estimating and subtracting the component due to the maternal ECG from the signals. Indeed, the maternal component is the main interference in abdominal fetal ECG recordings. Thus, mECG canceling procedure is the most common approach in fECG extraction and its robustness makes it a basilar in any non-invasive fECG analysis system. Maternal ECG canceling consists of the construction of an estimate of the mECG component and in its subtraction from the abdominal signals. Specifically, TS refers to a technique based on the estimation of the PQRST maternal pattern on the abdominal signals using its synchronization with the maternal QRS. Several TS techniques have been implemented in the literature, some are based on the construction of an average PQRST complex, which is then subtracted from each subsequent mECG after scaling and shifting operations [18,19], others apply PCA for dimensionality reduction [20,21,22]. 

In our methods, the mECG canceling procedure was performed estimating the approximation of each mECG beat by PCA implemented by SVD. First, to allow an accurate cancelling of mECG all signals obtained from the previous step were upsampled at 4 kHz with the Fourier transform method. Then, a trapezoidal window (whose length depends on the mean RR-interval computed on the whole record) is used to select and weight the signal around each detected mQRS. This operation allows obtaining weighted PQRST segments, which represent the columns of X, an nd×nq matrix, where nd is the length of the PQRST segments and nq is the number of mQRSs. This matrix is then decomposed using the “*thin*” form of SVD [44], which is valid for nd>nq, as follows:

(1) $$X=USV^{T}$$

where S is an nq×nq diagonal matrix of the singular-values, U *(*nd×nq*)* and V (nq×nq*)* are the unitary matrices of the left and right singular-vectors, respectively. The first columns of the matrix U, corresponding to the first eigenvectors, giving the largest contribution to covariance, likely represent the maternal PQRST waves. The matrix X is then rebuild (i.e., $X_{r}$) using a reduced number of singular-vectors:

(2) $$X_{r}=U_{r}S_{r}V_{r}^{T}$$

where the matrices $S_{r}$ (ne×ne), $U_{r}$ (nd × ne) and $V_{r}$ (nq × ne) contain “ne” number of singular eigenvalues and singular eigenvectors respectively. The final step consists in the subtraction of the estimated PQRST segments: they are first unweighted by the trapezoidal window and then the ending of each segment is connected with the beginning of following one with a straight line, obtaining an estimated mECG, which is then subtracted from the original signal.

For the FECGSYNDB, the two reference mECG channels were used to achieve a robust detection of maternal QRS complexes. For the PhysioNet CinC 2013 Database the component with the best mECG was identified by taking into account a priori knowledge of the QRS derivative, width and pseudo-periodicity. The maternal QRS detection was then performed on the selected ICA component. For a detailed description about maternal QRS detection see [23]. In both cases, maternal ECG canceling was performed independently for each of the four ICA separated channel.

**Figure 4:**
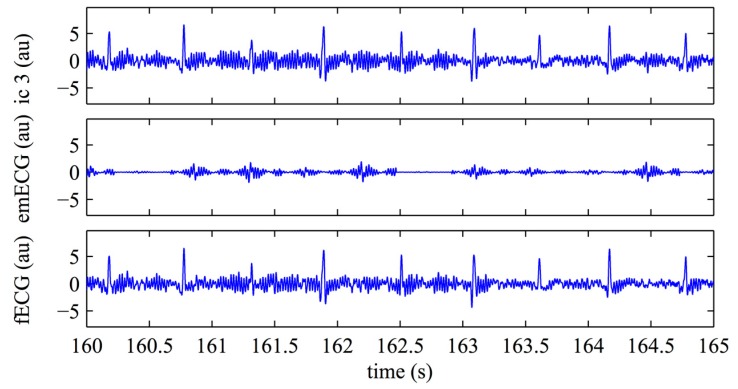
Application of maternal ECG canceling. Top: ECG after the application of ICA (ic3 in Figure 2); middle: estimated mECG (emECG) obtained by SVD; bottom: residual fECG signal resulting from by the two previous signals subtraction. Intensity values are expressed in arbitrary units (au).

Figure 4 shows the application of the mECG canceling to the independent component 3 (ic3) of Figure 3. In this example, the ICA step well separated the maternal and the fetal components so that the estimated maternal contribution is very small (Figure 4, middle) and canceling has a little added value: it removes small, maternal, residual spikes as that occurring at the time 164.5 s. This step removes mECG while leaving the noise.

#### 2.3.4. Fetal Quality Index Optimization

**Figure 5:**
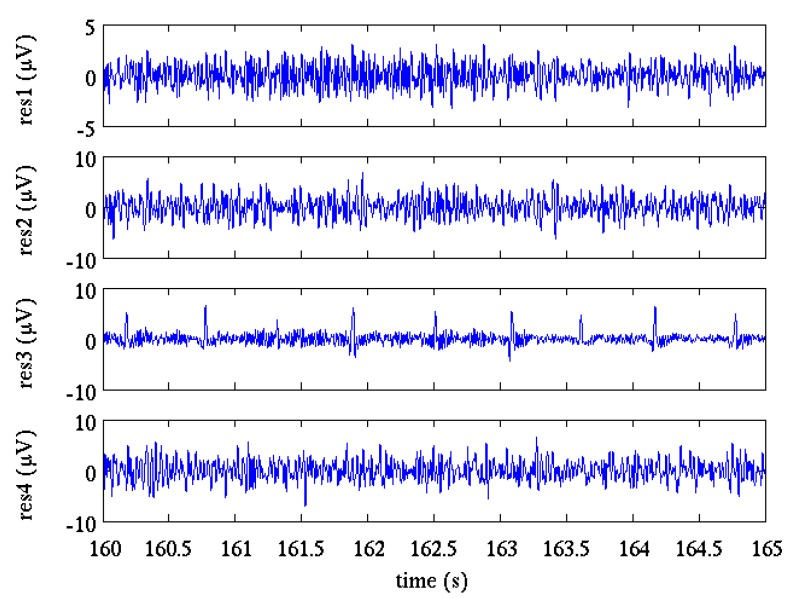
Residual signals (res1, res2, res3, res4) obtained after maternal ECG canceling from the four selected channels.

Maternal ECG canceling provides four residual signals (Figure 5), however the fECG amplitude could be still low compared to the other components or even not visible so the fQRS enhancement can increase the performance of the algorithm. 

In this step, a fQI, which characterizes the morphological and temporal characteristics of fECG is devised. The fQI is then computed on a generic signal obtained as linear combination of abdominal signals, thus resulting in a multivariate function of the coefficients of that linear combination. The algorithm finally attempts to maximize the fQI by searching for the maximum of this multivariate function. Thus, fQI is a value, between 0 and 1, which represents the quality of the fetal component estimated as linear combination of the abdominal signals. However, it should be noticed that fQI is specific of each record, in particular it is affected by fECG inter-beat interval. Therefore, the value of the maximum fQI is not an absolute index of quality of the enhanced fQRS signal extracted from the different abdominal ECGs, but is relative to each record.

In order to describe the characteristics of both the fECG and the noise, specific features were built which were based on signal derivatives and on specific time windows (see Table 2). For further details about the definition of these features see [34]. 

A fQI was then devised based on these features, the following empirical formulation was implemented:

(3) $$\mathrm{fQI}=\frac{\mathrm{Df}-\mathrm{Dn}-3*\mathrm{Dhn}-0.1*\mathrm{Dfa}-\varepsilon }{\mathrm{Df}+\mathrm{Dn}+3*\mathrm{Dhn}+0.1*\mathrm{Dfa}+\varepsilon }$$

in which ε is a very small constant introduced to avoid division by zero.

Once the fQI is devised, it is assumed that a linear combination of abdominal signals enhancing the fQRS exists. The fQI is computed on a generic signal *z* obtained by linear combination of the abdominal signals thus resulting in a multivariate function of the coefficients of such linear combination:

(4) $$z=a^{T}X_{r}$$

The aim of finding the signal *z* with maximum fQI can be achieved searching for the coefficients vector a which maximizes the function fQI(a). An analytic expression for the derivatives of this quality function does not exist, therefore direct search algorithms must be adopted in searching for the maxima. However, this function is scale independent (assuming the constant *ε* negligible), and an unconstrained optimization algorithm can be used. The Nelder-Mead algorithm [45] was selected as the optimization method as it gives good performance in low dimensional optimization problem (the number of abdominal signals is four) and represents a nice compromise between performance and convergence speed [38].

**Figure 6:**
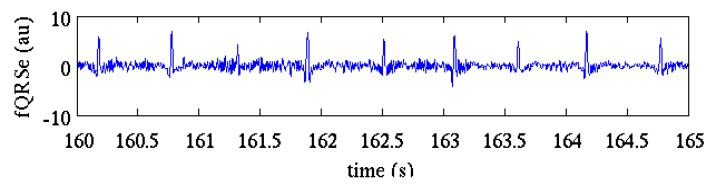
The fQRS-enhanced signal obtained by fQI optimization from the residual signals of Figure 5. Compared to the signal in Figure 4 (bottom) it can be observed that some QRSs (i.e., 161.4 s) are enhanced and the noise amplitude around the QRSs is lower. Arbitrary units are used for the ordinate axis because the optimization of fQI includes signals normalization.

Figure 6 shows the fQRS-enhanced signal extracted from the residual signals by the application of the fQI optimization algorithm. It can be observed how the application of the fQI optimization step improves the estimation of fECG compared to that obtained after ICA and canceling (Figure 4, bottom).

#### 2.3.5. Fetal QRS Detection

**Figure 7:**
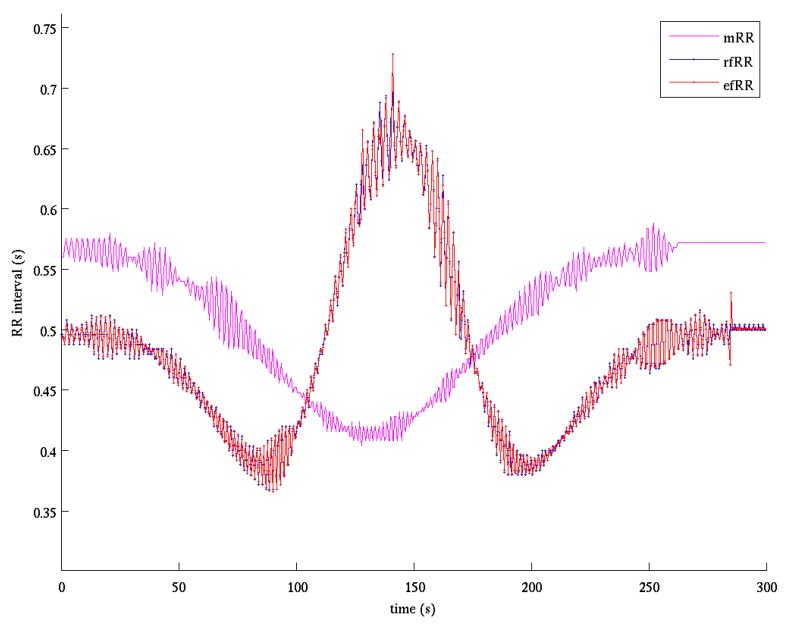
Fetal RR series estimation after the application of the ICAQIO-based method. mRR: maternal RR series obtained by the reference maternal channels (magenta); rfRR: reference fetal RR series (blue); efRR: estimated (red) fetal RR series obtained by the application of the two-pass fetal QRS detection procedure.

The procedure for fQRS detection was based on two passes. In the first one the absolute derivative of the enhanced fQRS was filtered by a forward-backward Butterworth bandpass filter (6.3–16 Hz). The QRS was then detected with an adaptive threshold on derivative amplitude automatically initialized and recursively updated depending on the temporal distance from the previous QRS detection [46]. The fiducial point of each detected QRS was selected as the time of the maximum or minimum (according to the sign assigned in the initialization phase) of the derivative signal. The second pass was based on a QRS detector, which, starting from the best fetal RR interval identified in the previous step, proceeded in forward/backward direction. Figure 7 shows an example of the estimated fetal RR series compared to the reference one. It is evident how the estimated series is almost superimposed to the reference one. It should be noted that the core of the QIO approach stands in finding the linear combination of signals that maximizes the fQI; for this reason, only one single fQRS component is extracted. Thus, QIO method is unsuitable to manage the twin pregnancy condition and for this reason we decided to exclude case 5 in the evaluation of the performances, as stated before.

**Figure 8:**
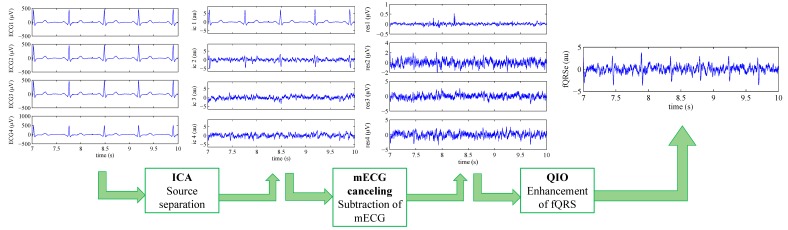
Application of the ICAQIO-based algorithm to the record “a75” of the PhysioNet CinC 2013 Database.

Figure 8 summarizes the main steps of the ICAQIO-based method in processing a real signal. In particular, we selected the record “a75” of the PhysioNet CinC 2013 Database.

### 2.4. Methods for Comparison

#### 2.4.1. QIO-Based Method

The QIO-based method depends uniquely by the capacity to enhance the fECG, after mECG canceling, on the basis of its temporal and morphological characteristics. 

As regards the FECGSYNDB, the method applied corresponds to that published in [34], with the exception of the mQRS enhancement and detection steps, as this database provide maternal reference. The method includes three steps: mECG canceling, enhancement of fQRS based on fQI optimization and fetal QRS detection (Figure 1). These modules are the same as described for the ICAQIO-based method. As discussed above for the ICAQIO-based approach, case 5 was not included in the evaluation of the performance of this algorithm.

As regards the PhysioNet CinC 2013 Database, which do not include the reference maternal channels, the algorithm included an additional step of mQRS enhancement, based on devising a mQI, and a maternal QRS detection step, as previously described in [34].

#### 2.4.2. ICA-Based Method

As regards the FECGSYNDB, the ICA-based method applied was a combination of mECG canceling and ICA for fECG separation. This method is a simplified version of the one presented by our team at the PhysioNet CinC 2013 [23,32]. Indeed, our CinC 2013 method applied a second step of ICA after mECG canceling which on the FECGSYNDB did not significantly improve the performance (data not shown). The approach used in this study includes the following three steps: separation of sources based on ICA, mECG canceling and fetal QRS detection (Figure 1). Also in this case, these modules are the same as described for the ICAQIO-based method. In particular, in the maternal ECG canceling step mECG channels were used for mQRS detection. For the application on the PhysioNet CinC 2013 Database, the original version of the approach, proposed in [23,32], was used.

The ICA-based approach applied the fQRS detection procedure to all the channels obtaining four hypothetical QRS annotations and the relative RR series. Then, the best estimated fQRS annotations (and so the best fECG channel) were automatically selected without considering the fQRS reference annotations. The selection was based both on the basis of the knowledge of the typical fetal RR values and on the minimization of a criterion based on the following features: the mean of absolute RR first derivative, the mean of absolute RR second derivative and the number of detected fQRSs matching maternal QRSs. The minimization of the mean of absolute RR first/second derivatives is based on the hypothesis that the fetal RR series is more regular respect to the one resulting from the application of the QRS detection algorithm to a noisy signal.

**Figure 9:**
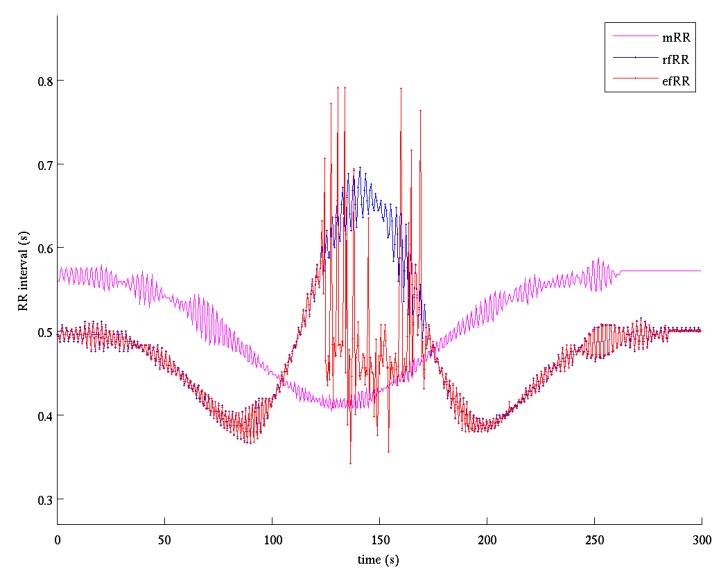
Fetal RR series estimation after the application of the ICA-based method. mRR: maternal RR series obtained by the reference maternal channels (magenta); rfRR: reference fetal RR series (blue); efRR: estimated (red) fetal RR series obtained by the application of the two-pass fetal QRS detection procedure.

Figure 9 shows the fetal RR series estimated by the ICA-based method for the same record of Figure 7. In the interval 130–170 s such estimate is bad as the ICA fails to sufficiently separate fetal component from noise and the performance is lower compared to the ICAQIO-based approach.

For the application on the FECGSYNDB, we implemented two different versions of the ICA-based method. The first one (ICA-based) was a complete algorithm applying an automatic channel selection for providing an estimated fetal RR series and an estimated fetal ECG. This method was compared with the ICAQIO-based and QIO-based methods on all the cases of the FECGSYNDB, excluding case 5 (Twin pregnancy). The second version of the algorithm (ICA-based_post) was aimed at comparing the performance of our algorithm with the ICA methods tested in [35], which were applied on the same FECGSYNDB. These methods all selected the channel whose estimated fQRS annotations best fitted with the reference annotations, so the ICA-based_post algorithm also was implemented in this way, without the automatic choice of the channel. To compare our algorithm with those tested in [35], we applied the ICA-based_post on all the cases of the FECGSYNDB, including case 5 and considering all the noise levels. 

### 2.5. Evaluation of fQRS Detection

The performance of the tested methods was evaluated on the total length of the fQRS signal, using sensitivity (*SE*), positive predictive accuracy (PPA) [47] and their harmonic mean (*F*1) [14]:

(5) $$SE=\frac{TP}{TP+FN}$$

(6) $$PPA=\frac{TP}{TP+FP}$$

(7) $$F1=2\cdot \frac{PPA\cdot SE}{PPA+SE}=\frac{2\cdot TP}{2\cdot TP+FN+FP}$$

where *TP* indicates the number of true positives (correctly detected fQRS), *FN* the number of false negatives (missed fQRS detections) and *FP* the number of false positives (falsely detected non-existent fQRS). For the calculation of *SE* and *PPA*, each fQRS detection was considered correct if it differed of less than 50 ms from the reference annotation. Since the first and last fQRSs could be sometimes mis-annotated, they were excluded from the evaluation. 

To test the significant differences within the different cases we reported *F*1 gross values for the different cases and applied the McNemar test on paired proportions for evaluating paired differences between methods.

## 3. Results

### 3.1. Simulated Data: FECGSYNDB

The overall gross statistics, which is obtained by computing the *F*1 for all the records with the two lowest level of SNR (excluding case 5), showed that the proposed combined ICAQIO-based algorithm obtained the best performance (98.78%) over both the single methods (QIO-based: 97.77%; ICA-based: 97.61%). As regards the two single methods, the QIO-based method outperformed the ICA-based one. 

Moreover, the ICA-based_post algorithm, with a posteriori selection of the channel, for comparison with the previously ICA tested algorithms on the FECGSYNDB in [35], obtained a performance of 97.51%. 

In Table 3 the gross values of *F*1 for the ICAQIO-based method, the QIO-based method and the ICA-based method for each case are reported.

**Figure 10:**
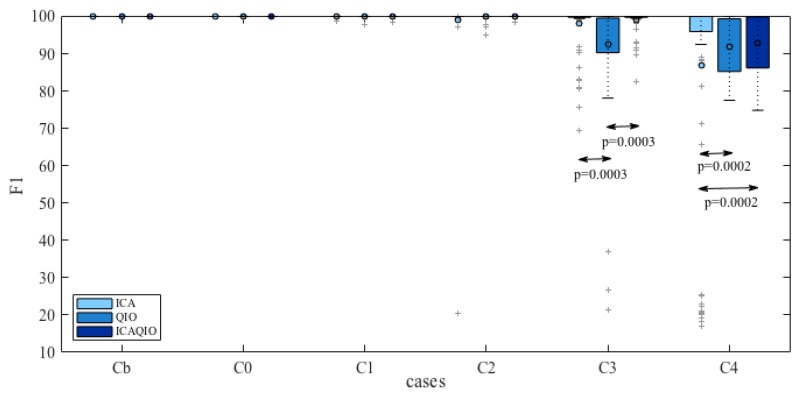
Comparison of *F*1 results for the three tested algorithms, ICAQIO-based, QIO-based and ICA-based, all the selected cases. Dots represent mean values, crosses represent extreme values. Samples tested: 1750 synthetic signals; 0 and 3 dB SNR levels; 10 different heart dipole models; 5 simulations.

Comparing paired differences within each case between the performance of the ICAQIO-based, the QIO-based and the ICA-based algorithms, we obtained that in case 3 the *F*1 gross value of the ICAQIO-based method was significantly higher than that of the QIO-based method (*p* = 0.0003) and the *F*1 gross value of ICA-based method was significantly higher than that of QIO-based method (*p* = 0.0003). No significant differences between the ICAQIO-based method and the ICA-based method were obtained in case 3. In case 4 the *F*1 gross value of the ICAQIO-based method was significantly higher than that of the ICA-based method (*p* = 0.0002) and the *F*1 gross value of QIO-based method was significantly higher than that of ICA-based method (*p* = 0.0002). No significant differences between the ICAQIO-based method and the QIO-based method were obtained in case 4. No significant differences among the three methods were obtained in the other cases. Figure 10 shows the F1 of the three methods for all the cases.

**Figure 11:**
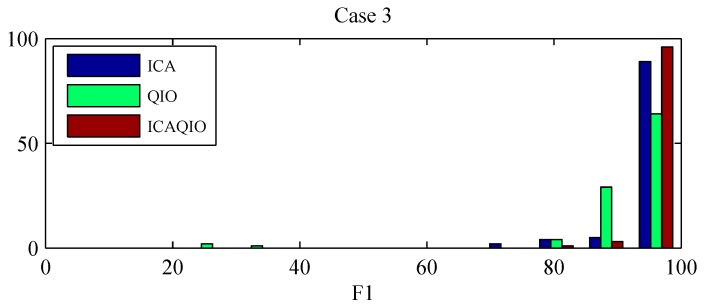
*F*1 histograms for the three tested algorithms, ICAQIO-based, QIO-based and ICA-based, for case 3 (uterine contraction). Samples tested: 1750 synthetic signals; 0 and 3 dB SNR levels; 10 different heart dipole models; 5 simulations.

**Figure 12:**
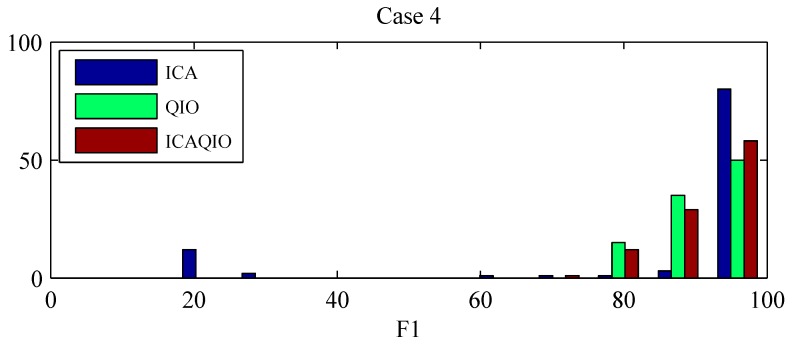
*F*1 histograms for the three tested algorithms, ICAQIO-based, QIO-based and ICA-based, for case 4 (maternal and fetal ectopic beats + noise). Samples tested: 1750 synthetic signals; 0 and 3 dB SNR levels; 10 different heart dipole models; 5 simulations.

Figure 11 and Figure 12 show the histograms of *F*1 for each method for the cases in which statistically significant differences were found among the different methods, i.e., case 3 and case 4. It can be observed that the distributions are skewed and there are records for which the methods fail in giving acceptable performance. In particular, the ICA-based method, has more records for which it gives a very low performance. For this reason, the gross values of *F*1, along with the min and the max values, are reported in Table 3 as a representative measure of the overall performance of the algorithms. 

### 3.2. Real Data: PhysioNet CinC 2013 Database

The gross F1 score of the proposed ICAQIO-based algorithm, which was obtained by computing the value for all the selected records of the PhysioNet CinC 2013 Database, was 99.38%.

This performance was slightly higher than that obtained for the ICA-based method (99.37%), which was the method implemented for the PhysioNet CinC 2013 with the few changes as explained in [34]. On this Database, the QIO-based method was the one that obtained the highest performance with a gross *F*1-value of 99.76% [34]. The performance obtained by the three different algorithms are summarized in Table 4.

## 4. Discussion

In this paper, we propose a novel hybrid fetal extraction algorithm, which combines the classical mECG canceling procedure with two other different approaches for fetal QRS enhancement: independent component analysis and a quality index optimization criterion. This algorithm integrates two methods that we previously developed and tested on the PhysioNet CinC 2013 Database. The first one is a method based on ICA, which is a simplification of the method that we presented at the Physionet CinC 2013 [23,32]. Indeed, in the implementation on the FECGSYNDB, with respect to the previous algorithm, we eliminated the second step of ICA as we observed that, on this database, it did not improve the performance (data not shown). The second method is an algorithm based on devising a quality index that is built exploiting the morphological and temporal characteristics of the signal of interest (in this case the fECG) and then finding a linear combination of signals which maximizes this index [34]. It should be noted that all the methods integrate a classical step performing mECG canceling, aimed at removing from the abdominal mixture this undesired component before obtaining the final fECG. Indeed, in our previous paper [34] we have shown as the mECG canceling is fundamental and that its integration with ICA provides better results than ICA alone. Moreover, QIO optimization alone is not able to separate the fECG from the mECG.

Considering the overall gross statistics, according to our expectations, we found that on the FECGSYNDB the combined ICAQIO-based method outperformed the two methods applied as single (*F*1 ICAQIO-based: 98.78%; *F*1 QIO-based: 97.77%; *F*1 ICA-based: 97.61%). This can be due to the fact that the two methods use different criteria to enhance the fQRS so that one can succeed when the other fails. Indeed, in our previous paper [34], we have compared the performance of ICA-based and QIO-based methods record-by-record and we observed that, although generally the QIO-based method outperformed ICA-based one, for some records, the opposite was found. Thus, as we have hypothesized, a combination of the two approaches improves the overall performance in fQRS detection. The improvement obtained adding a successive step of fQIO optimization after ICA and mECG canceling, can be observed from the comparison of fECG signals in Figure 4 (bottom trace) and 6 and even more from the comparison of the estimated fetal RR series shown in Figure 7 and Figure 9. Indeed, in the represented record, while the application of ICA + mECG canceling (ICA-based method) presents some errors in the estimation of the fetal RR series, the fetal RR series estimated with the ICAQIO-based approach is exactly superimposed to the reference series. 

On the real data of the PhysioNet CinC 2013 all the three methods performed very high, above 99%, without a significant difference among the three methods. The performance of the ICAQIO-based approach was slightly higher than that of ICA-based method, the performance of the QIO-based method slightly exceeded that of ICAQIO-based approach. 

It should be highlighted that both the ICA-based and QIO-based methods were developed using the “set-a” of the PhysioNet CinC 2013 as learning set using some tuning of the algorithms on these data to maximize the performance. For example, the optimal type of ICA algorithm, contrast function and number of eigenvalues were selected for the ICA-based method, while the coefficients and window lengths were chosen for the QIO-based method. Despite this tuning, the two methods have demonstrated to be highly generalizable, giving still high performance, although a bit lower to that obtained on the “set-a” of the Challenge, on other datasets. Indeed, the ICA-based obtain the top official score on the “hidden” set of the Challenge and both the methods performed overall above 97% on the FECGSYNDB.

This tuning could however have led to an over-estimation of the performances of the ICA-based and the QIO-based methods on this specific dataset, making less evident the improvement of the combination of the two criteria, were the performance of combined method was overall comparable to the ICA-based approach and the QIO-based method. 

When applied on larger database including several non-stationary and critical conditions for fECG extraction, the ICA-based and QIO based approaches, while still giving an overall high performance, failed for some records and gave lot of extreme values. The ICAQIO-based on the contrary, limited the number of extreme values, highlighting the advantage of combining the criteria of independence of sources and quality index optimization.

Considering the ICA-based and the QIO-based method separately, we observed that the QIO-based approach obtained a higher score than that of the ICA-based with an a priori selection of the channel (ICA-based), both on the FECGSYNDB and on the PhysioNet CinC 2013 Database. This result confirmed our previous findings [34]. The better performance obtained by the proposed QIO-based approach could be due to some several reasons. First, the ICA-based approaches can fail in separating fECG if the number of underlying sources is higher than the number of the measured signals and if the fECG power is small compared to noise. In addition, the QIO-based method eliminates the problem of ICA-based approach of automatically selecting the mECG (or fECG) among the estimated independent sources. 

Comparing the performances separately for each case on the FECGSYNDB, we observed that the most significant differences were obtained for case 3 and case 4, that were uterine contraction and maternal and fetal ectopic beats + noise, respectively. Thus, in these cases the combinations of the two criteria, ICA and fQI optimization, can be particularly helpful in the accurate extraction of fECG. We presented gross *F*1 values for each case to have a more general evaluation of the performance of the algorithms. Indeed, the distributions of the *F*1 values are skewed, with several records giving high performance but other records for which the performance is low, i.e., extreme values. In particular, in case 3 the QIO-based method presents more low-performance records, while in case 4 the ICA-based method has more extreme values. Indeed, in case 3 *F*1 value of the ICAQIO-based method was significantly higher compared with that of QIO-based method and in case 4 with that of ICA-based method. In this sense, the ICAQIO-based approach, which combines the two criteria, is overall more robust to extreme values compared to both ICA-based and QIO-based algorithm in particular in critical conditions as uterine contractions and ectopic beats. 

In general, it should be observed that for all the three compared methods (ICAQIO-based, QIO-based and ICA-based) we obtained high gross overall *F*1 scores, which were all above 97% on the FECGSYNDB and above 99% on the PhysioNet CinC 2013 Database. Notably these results were obtained considering the lowest SNR levels, thus showing that the methods proposed are robust to high noise level. Importantly the proposed methods work in a fully unsupervised way with the same parameter setting for all the selected records of the database.

In this study, we also tested the ICA-based method with a posteriori selection (ICA-based_post), in order to compare the performance of our algorithm on the FECGSYNDB, to that of the methods tested in [35]. We obtained a performance of 97.51%, which was slightly higher than the one obtaining the best performance using JADE in [35] (97.46%). These results confirm that our hybrid approach based on the combination of ICA and mECG cancelling outperformed the results of the most common BSS-based methods for fECG extraction.

Some limitations of the proposed method should be considered. First, the algorithms were only tested on simulated data and on the Physionet Challenge 2013 Database, with a possible over-estimation of the results. Further tests on realistic data are needed to better evaluate the performance of the different algorithms and to evaluate their robustness as well as their ability to extract fECG in different recording conditions such i.e., ectopic beats and uterine contractions. In particular, systematic tests of the performance of the technique as a function of changes in realistic noise, signal quality of mECG, relative amplitude and signal quality of fECG, number of channels of mECG and fECG, nonstationarty, nonstationary mixing and arrhythmia are needed. 

Another drawback is the computation time of the proposed algorithm, which was about one minute for each record of 5 minute duration with Matlab R2014a on a Samsung NP730U3E Notebook (i7-3537U, 2 GHz x4; DDR3 6 GB—1600 MHz; SSD 256GB; Linux Ubuntu 16.04, Samsung, Seoul, South Korea ). A decrease of the computation time could be achieved both using a more efficient or more suitable optimization algorithm and/or tuning/changing the fQI function. However, this optimization could be possible, without truly incurring in over-fitting, only if a larger annotated database were available. Finally, the QIO approach is based on finding the linear combination of signals that maximizes the fQI, thus only one single fQRS component is extracted. For this reason, the QIO method, and so the combined ICAQIO-based method, is unsuitable to manage a twin pregnancy condition. In the future, the algorithm could be modified in order to also manage this condition thus allowing the extraction of two fECG components. One possible solution could be to use multiple QIO algorithms with an ICA module at each iteration, thus integrating the search for the maximum of fetal QI with the search for the maximum independence.

## 5. Conclusions

In this paper, we have introduced a novel combined independent source separation and quality index optimization method (ICAQIO-based) for fECG extraction from abdominal maternal leads. The method was tested and compared with the two single methods, on the recently developed FECGSYNDB for benchmarking of fECG extraction and detection algorithms and on the PhysioNet CinC 2013 Database. The comparison of the three methods showed that the combination of the criterion of independence of source and the optimization of a fetal quality index optimization, outperformed the two methods applied alone, in particular in critical conditions like uterine contraction and maternal and fetal ectopic beats, where the two criteria applied independently give more low performance records. The algorithm can be applied in a fully unsupervised way and also works in the presence of low amplitude fECG signals and noise. Future studies will be needed to test the performances of the algorithm in a real-world scenario with different conditions of noise, recording setup and fetal configurations.

## Figures and Tables

**Table 1:** Description of the seven cases generated to simulate different physiological events.

Case	Description
Baseline	Abdominal mixture of fECG and mECG without noise or events
Case 0	Baseline + noise (no events)
Case 1	Fetal movement + noise
Case 2	mHR/fHR acceleration or deceleration + noise
Case 3	Uterine contraction
Case 4	Maternal and fetal ectopic beats + noise
Case 5	Twin pregnancy + noise

fECG = fetal electrocardiogram; mECG = maternal electrocardiogram, mHR = maternal heart rate, fHR = fetal heart rate.

**Table 2:** Quantities and features defined for devising the fQI.

**Quantities Characterizing fECG and Noise**	
adf	absolute value of the derivative computed on short intervals (0.013 s), which enhances the fQRS and smoothes the noise
adhn	absolute value of the derivative computed on shorter intervals (0.003 s), which takes into account mostly the noise
w04	window of 0.4 s specific for the fQRS
w013	window of 0.13 s for high frequency noise
w01	window of 0.1 s for very high frequency noise
w40	window of 4.0 s for isolated impulsive artefacts
**Features based on the above quantities**	
Df	trimmed mean of the maxima of adf computed on successive w04
Dn	trimmed mean of the maxima of adf computed on successive w013
Dhn	trimmed mean of the maxima of adhn computed on successive w01
Dfa	trimmed mean of the maxima of adf computed on successive w40

**Table 3:** F1 % results for each case and each method, shown as gross value (min, max) ^1^.

Method	Baseline	Case 0	Case 1	Case 2	Case 3	Case 4
ICA-based	100.00 (100.00, 100.00)	100.00 (100.00, 100.00)	99.98 (98.70, 100)	99.17 (20.50, 100.00)	98.27 (69.60, 100.00)	86.62 (17.00, 100.00)
QIO-based	100.00 (100.00, 100.00)	100.00 (99.80, 100.00)	99.97 (97.70, 100.00)	99.88 (95.00, 100.00)	92.75 (21.50, 100.00)	92.59 (77.50, 100.00)
ICAQIO-based	100.00 (99.90, 100.00)	100.00 (100.00, 100.00)	99.97 (98.30, 100.00)	99.97 (98.50, 100.00)	99.26 (82.70, 100.00)	93.68 (74.80, 100.00)

^1^ Values are obtained for SNR = 0 and SNR = 3 dB.

**Table 4:** Gross statistics obtained for each method shown as % (min, max).

Method	*SE*	*PPA*	*F*1
ICA-based	99.36 (87.97, 100.00)	99.38 (89.06, 100.00)	99.37 (88.81, 100.00)
QIO-based	99.75 (96.62, 100.00)	99.76 (96.62, 100.00)	99.76 (96.62, 100.00)
ICAQIO-based	99.38 (89.84, 100.00)	99.38 (89.84, 100.00)	99.38 (89.94, 100.00)

## Abbreviations

The following abbreviations are used in the manuscript.

BSS	Blind source separation
CHD	Congenital heart disease
fECG	Fetal electrocardiogram
FHR	Fetal heart rate
ICA	Independent Component Analysis
mECG	Maternal electrocardiogram
PCA	Principal Component Analysis
PPA	Positive predictive accuracy
QI	Quality index
QIO	Quality index optimization
SE	Sensitivity
SNR	Signal to noise ratio
SVD	Singular Value Decomposition
TS	Template subtraction
